# Supplementary material for: What’s for dinner? Diet and potential trophic impact of an invasive anuran Hoplobatrachus tigerinus on the Andaman archipelago
Source: PeerJ. 2018 Oct 2;6:e5698. doi: 10.7717/peerj.5698 (PMC6173161; doi:10.7717/peerj.5698)
Supplement: Supplemental Information 1 — Sampling carried out using pitfall traps in four habitat types, over two seasons in three sites of the Andaman archipelago. [file peerj-06-5698-s001.docx]

**Supplemental Material 1.** Simpson’s index of diversity for terrestrial invertebrate prey, sampled using pitfall traps in four habitat types, over two seasons.

|  | **Agriculture** | | **Plantation** | | **Disturbed Forest** | | **Undisturbed Forest** | |
| --- | --- | --- | --- | --- | --- | --- | --- | --- |
|  | dry | wet | dry | wet | dry | wet | dry | wet |
| Karmatang | 2.67 | 3.82 | 3.82 | 4.95 | 1.93 | 4.20 | 1.66 | 3.65 |
| Webi | 3.89 | 3.95 | 2.91 | 2.71 | 3.79 | 4.74 | 2.15 | 4.81 |
| Wandoor | 2.28 | 2.25 | 2.04 | 5.38 | NA | 6.13 | NA | 4.74 |
